# Potential of Pterostilbene as an Antioxidant Therapy for Delaying Retinal Damage in Diabetic Retinopathy

**DOI:** 10.3390/antiox14030244

**Published:** 2025-02-20

**Authors:** Raquel Burggraaf-Sánchez de las Matas, Isabel Torres-Cuevas, Iván Millán, María del Carmen Desco, Candela Oblaré-Delgado, Miguel Asensi, Salvador Mena-Mollá, Camille Oger, Jean-Marie Galano, Thierry Durand, Ángel Luis Ortega

**Affiliations:** 1 Hospital of Sagunto, Ramón y Cajal Av. s/n, 46520 Sagunto, Spain; burggraaf_raq@gva.es; 2 Department of Physiology, Faculty of Pharmacy, University of Valencia, Vicente Andrés Estellés Av. s/n, 46100 Burjassot, Spain; maria.i.torres@uv.es (I.T.-C.); canodel@alumni.uv.es (C.O.-D.); miguel.a.asensi@uv.es (M.A.); salvador.mena@uv.es (S.M.-M.); 3 Cavanilles Institute of Biodiversity and Evolutionary Biology, University of Valencia, Catedrático José Beltrán Martínez st, 46980 Paterna, Spain; ivan.millan-yanez@uv.es; 4 Vitreo-Retina Unit, Fundación de Oftalmología Médica de la Comunidad Valenciana (FOM), Pío Baroja st 12, 46015 Valencia, Spain; carmen.desco@uchceu.es; 5 Department of Medicine and Surgery, Faculty of Health sciences, Universidad CEU Cardenal Herrera, Luis Vives st 1, 46115 Alfara del Patriarca, Spain; 6 Institut des Biomolécules Max Mousseron (IBMM), Pôle Chimie Balard Recherche, UMR 5247, Université de Montpellier, CNRS, ENSCM, 340093 Montpellier, France; camille.oger@umontpellier.fr (C.O.); jean-marie.galano@umontpellier.fr (J.-M.G.); thierry.durand@umontpellier.fr (T.D.)

**Keywords:** diabetic retinopathy, lipid peroxidation, oxidative stress, polyphenols, pterostilbene, antioxidant therapy

## Abstract

Chronic hyperglycemia is a major driver of neurovascular damage in diabetic retinopathy (DR), a leading cause of preventable blindness in adults. DR progression is often undetected until its advanced stages, with oxidative stress recognized as a primary contributor. In diabetes, oxidative stress disrupts retinal cellular balance, damaging proteins, DNA, and lipids, and triggering photoreceptor degeneration. Pterostilbene (Pter), a polyphenol with antioxidant properties, has demonstrated protective effects in DR animal models and was assessed in a pilot clinical study. DR patients treated with 250 mg/day of oral Pter showed a reduction in the development of retinal vascular alterations characteristic of the disease. Urinary analyses confirmed Pter’s role in reducing the lipid peroxidation of polyunsaturated fatty acids (PUFAs), including arachidonic and adrenic acids, indicators of oxidative damage in DR. Pter also improved the GSH/GSSG ratio, reflecting a restored redox balance. However, after six months without treatment, retinal damage indicators reappeared, highlighting the importance of sustained intervention. These findings suggest that Pter may help slow the progression of DR by protecting against oxidative stress and highlight the importance of implementing antioxidant therapies from the diagnosis of diabetes, although its long-term impact and the development of consistent biomarkers deserve more research to optimize DR management.

## 1. Introduction

The prevalence of diabetes is continuously increasing worldwide. Currently, approximately 529 million people suffer this pathology, and it has become one of the main causes of death and disability around the world [1]. The complications derived from chronic hyperglycemia cover a wide spectrum ranging from a decreased quality of life to the development of lethal effects. At the ocular level, diabetic retinopathy (DR) is the main cause of preventable blindness in people of working age [2,3]. DR is an asymptomatic pathology at the beginning, which means that it can be found in advanced stages when it is diagnosed. The results from a multicenter Early Treatment of Diabetic Retinopathy Study (ETDRS) [4,5] established the fundamental clinical classification criteria for DR. According to these criteria, it can be mainly classified into two clinical forms, non-proliferative DR (NPDR) and proliferative DR (PDR), based on both the ophthalmic lesions observed and the formation of new atypical vessels [6,7]. Diabetic macular edema (DME) is the main cause of blindness in diabetic patients and, although it can appear at any stage, is more common in the advanced stages of the disease [5]. Immunotherapy and laser photocoagulation are therapies against DR; however, their use is limited to its advanced stages, when neurovascular retinal damage induced by the disease cannot be reversed. This is the main reason to search for alternative therapies that can be used before the appearance of irreparable injuries.

Poor glycemic control, reflected in elevated HbA1c levels, as well as the duration of diabetes, arterial hypertension, dyslipidemia, and a high body mass index caused by hyperglycemia, are recognized as key risk factors for DR [8,9,10,11]. Due to the multifactorial origin of DR, a precise pathophysiological understanding of the development and progression of DR remains unclear. However, there is a broad consensus when considering the oxidative stress produced by chronic hyperglycemia as a crucial factor involved in the disease, which produces elevated levels of lipid peroxidation, protein carbonylation, and free radicals [5,7,12,13,14,15,16,17]. The alteration in redox homeostasis induces retinal neurovascular impairment, including neuronal and glial degeneration, vascular dysfunction, a thickening of the vascular basement membrane, a loss of pericytes and endothelial cells, a disruption of the inner blood–retinal barrier, and finally, PDR and DME development [14].

Polyphenols constitute a heterogeneous family of plant secondary metabolites with antioxidant and anti-inflammatory properties. The health benefits of these bioactive molecules have been demonstrated in the prevention and treatment of different chronic injuries related to oxidation, such as diabetes, high blood pressure, or neurodegenerative diseases [18,19]. Pterostilbene (trans-3,5-dimethoxy-4′-hydroxystilbene) (Pter), a structural analog of resveratrol, presents better intestinal absorption, elevates hepatic stability, and has higher bioavailability than other stilbenes [20,21]. We have demonstrated in an animal model that the antioxidant properties of Pter protect against early neuro-retinal damage caused by hyperglycemia [13] and prevents retinal early lipid peroxidation [12], a determinant process in the development and evolution of DR. Based on this evidence, we hypothesize that Pter treatment is a promising approach to delay the natural progression of DR to advanced stages in patients.

## 2. Materials and Methods

### 2.1. Study Population, Design, and Informed Consent Statement

This research was a single-center, randomized, placebo-controlled pilot study involving 30 patients with type 2 diabetes *mellitus*, mild to moderate NPDR, and non-clinically significant DME. The Spanish Agency of Medicines and Medical Devices (AEMPS) confirmed that the current study was not classified as a clinical trial involving medicines. Therefore, no specific authorization was required for this classification. Nonetheless, approval from at least a local ethics committee was deemed mandatory. The subjects were recruited following a convenience sampling according to the inclusion and exclusion criteria from November 2016 to December 2022 at the Medical Ophthalmology Foundation (FOM; Valencia, Spain) (Table 1). We obtained approval from a local ethics committee (Foundation for the Promotion of Health and Biomedical Research in the Valencian Community) with ID (PI_63) and from the Human Research Ethics Committee (University of Valencia) (ID 1892235). Informed consent was obtained from all subjects involved in this study after carefully reading and understanding the information brochure at their screening visit.

Patients were randomly assigned to the treatment (Pter) or the placebo group (control). The basal DR stage was determined following the ETDRS grading system [4], and the presence of non-clinically significant DME was analyzed with optical coherence tomography (OCT). There were 4 post-randomization losses. Two participants from the treatment group dropped out due to their inability to accomplish the required visits. One participant from the placebo group died during follow-up from a cardiovascular event, and one subject was withdrawn due to non-compliance with the protocol. Hence, the final sample included 13 patients in the placebo group and 13 patients in the treatment group. The experimental determinations were carried out sequentially over time.

### 2.2. Treatment and Placebo Supplementation

Patients were supplemented for a period of 12 months. The intervention group received 250 mg of Pter daily in two doses (125 mg each) using the commercialized formula PteroPure^®^ (Life Extension, Lauderdale Lakes, FL, USA). This dose was chosen because it was well tolerated according to a previous safety study [22]. To improve compliance, the contents of 5 tablets were mixed in a packet, and patients were instructed to pour the contents into a glass of water or plain yogurt. The placebo group received 250 mg of microcrystalline cellulose daily, harmless to blood glucose levels, also given in a similar packet.

### 2.3. Study Protocol and Visits

This study spanned 18 months, with supplementation administered during the first 12 months, followed by an additional 6-month period to assess outcomes. The study protocol included 7 visits. Table 2 shows the measurements and actions performed at each visit. Visits 0, 2, 3, and 4 were used to deliver the supplement and check treatment adherence by asking patients to return empty packets.

The body mass index (BMI) was calculated by measuring the weight in kilograms (kg) with a digital scale and the height in meters (m) of the subjects, using the following formula:

BMI = kg/m^2^

Blood pressure (BP) (mmHg) was determined with a digital sphygmomanometer. To exclude a possible white coat syndrome, at least 3 measurements were taken at each visit, and the average between the three was taken into account.

The best corrected visual acuity (BCVA) was determined using the optotype designed for the ETDRS, obtaining as a value a number of letters with a range between 34 (according to the inclusion criteria) and 100.

Intraocular pressure (IOP) (mmHg) was measured with an air puff automated tonometer (Topcon^®^, Tokyo, Japan).

We used the macular OCT software (Topcon^®^, IMAGEnet 6 software, version V 1.31) to measure the central macular thickness (CMT) (μm).

Grade of DR: we performed a wide-field fundus retinography and fluorescein angiography with the OPTOMAP^®^ (Scotland, UK) device at T0, T1, and T2. The retinographies and fluorescein angiographies were analyzed by two trained ophthalmologists to determine the stage of DR according to the ETDRS system [4]. Dotted hemorrhages were considered mild, moderate were those with a size less than 1/3 of the optic disk diameter, and severe were those with a size greater than 1/3 of the optic disk diameter. The percentage of agreement reached was 96%. A third evaluator was asked in case of disagreement.

The venous tortuosity index (VTI) was calculated using the mathematical code developed by Khansari et al. [23]. This code allows the isolation of the vascular tree from color retinographies, obtaining images with the vessels segmented in binary format. The VTI of the 4 major veins was calculated using MATLAB software, version R2021b (Natick, MA, USA).

### 2.4. Clinical Blood and Urine Sample Analysis

Blood and urine samples were collected after an overnight fast. For each patient, a blood sample was collected into two plastic tubes with ethylenediaminetetraacetic acid disodium salt (EDTA, 1.8 mg/mL) and stored at −80 °C until use. HbA1c (%) was analyzed by reversed-phase cation exchange high performance liquid chromatography using the Adams A1C HA-8180V instrument (ARKRAY, Inc., Kyoto, Japan). On the other hand, the blood glucose levels, lipid profile, liver profile, and creatinine in the urine and blood were determined using the AU 5800 spectrophotometer Beckman Coulter instrument (Beckman Coulter, Brea, CA, USA).

### 2.5. Lipid Peroxidation Analysis in Urine

After thawing on ice, 1 mL of urine was collected and 5 µL of internal standard (PI, PGF_2α_-D4 at 20 µM) was added to each sample to give a final concentration of 500 nM. To remove solid residues, the samples were centrifuged at 14,000× *g* for 10 min at 4 °C. The supernatants were used for the clean-up and preconcentration step by solid phase extraction (SPE) using Discovery^®^ DSC-18 SPE 96-well plates (Sigma-Aldrich, St. Louis, MO, USA) and a PlatePrep 96-well Vacuum Manifold (Sigma-Aldrich, St. Louis, MO, USA). The SPE cartridges were initially conditioned with 1 mL CH_3_OH and 1 mL H_2_O (pH 3, 0.1% *v*/*v* HCOOH). The samples were then added to the SPE wells, and each well was washed with 500 µL of H_2_O (pH 3, 0.1% *v*/*v* HCOOH) and 500 µL of heptane.

The samples were eluted with 4 fractions of 100 µL of ethyl acetate in 96-well sample plates (Acquisty UPLC 700 L, from Waters, Barcelona, Spain). The recovered extracts were evaporated in a SpeedVac™ (Savant SPD111V, Thermo Scientific, Waltham, MA, USA) at 45 °C. Finally, the dried residue was resuspended in 60 µL of a solution of H_2_O (pH 3, 0.1% *v*/*v* HCOOH):CH_3_OH (85:15 *v*/*v*) before proceeding to analysis by UPLC-MS/MS.

UPLC-MS/MS analysis was carried out on an Qtrap 6500 plus (Sciex, Framingham, MA, USA). The conditions used were as follows: ionization in negative mode (ESI-); capillary voltage: 4.5 kV; source temperature: 475 °C; CUR (curtain gas): 35 psi; GS1 (ionization gas) and GS2 (auxiliary gas): 60 psi.

Separation conditions were selected to achieve appropriate chromatographic retention and resolution by using a C18 column (2.1 × 100 mm, 1.7 μm) from Phenomenex. A binary mobile phase CH_3_OH-CH_3_CN (50:50):H_2_O (0.1% *v*/*v* HCOOH) with gradient elution was used. The flow rate was 0.3 mL/min, and the temperatures of the column and the autosampler were 30 °C and 4 °C, respectively. The injection volume was 5 µL. The gradient started with 10% *v*/*v* CH_3_OH-CH_3_CN (i.e., channel B), and from 0 to 4.0 min, % B increased up to 90%. Finally, the mobile phase composition returned to the initial conditions at 9 min, and it was maintained for 12 min for system conditioning.

The detection was performed by multiple reaction monitoring using the acquisition parameters obtained in a previous work [12].

For data acquisition and processing, the SCIEX OS-MQ software from SCIEX (Framingham, MA, USA) (https://sciex.com/products/software/sciex-os-software, accessed on 1 April 2024) was used. The results were standardized by the creatinine levels, measured using a creatinine assay (MicroVue creatinine EIA, Quidel Corporation, San Diego, CA, USA) according to the manufacturer’s instructions.

The standards PGE_2_, PGF_2α_, 15-E_2t_-IsoP, 15-F_2t_-IsoP, 15-keto-15-E_2t_-IsoP, 5-F_2c_-IsoP, 2,3-dinor-15-epi-15-F_2t_-isoP, 15-keto-15-F_2t_-IsoP and deuterated internal standard PGF_2α_-d_4_ were obtained from Cayman Chemical Company (Ann Arbor, MI, USA).

The standards 10-epi-10-F_4t_-NeuroP, 4(*RS*)-4-F_4t_-NeuroP, 14(*RS*)-14-F_4t_-NeuroP, 17-F_2t_-dihomo-IsoP, *Ent*-7(*RS*)-7-F_2t_-dihomo-IsoP, 17-epi-17-F_2t_-dihomo-IsoP, 17(*RS*)-10-epi-SC-Δ^15^-11-dihomo-IsoF, 7(*RS*)-ST-Δ^8^-11-dihomo-IsoF, 5-F_2t_-IsoP were synthesized and validated at the Institute of Biomolecules Max Mousseron (IBMM) (Montpellier, France) by Professor Durand’s team [24].

### 2.6. Redox Pairs and Transsulfuration Pathway Metabolites by UPLC-MS/MS Analysis

GSSG, GSH, cystine, cysteine, homocysteine, homocysteine, and γ-glutamylcysteine, cystathionine, methionine, SAM, and SAH were extracted from blood samples (200 µL) treated with 10 mM N-ethylmaleimide (NEM) (200 µL) for 1 min. Then, perchloric acid was added to obtain a 6% concentration and centrifuged at 15,000× *g* for 15 min at 4 °C. The concentration of the analytes was determined in the supernatants by UPLC-MS/MS. This method was performed using the protocol of Escobar et al. [25].

### 2.7. Statistical Analyses

The data obtained in all experiments were presented as average ± standard deviation. If the data were normally distributed and had equal variances, a one-way ANOVA was employed to determine the differences among groups, followed by Tukey’s multiple comparison test. Otherwise, a non-parametric test, the Mann–Whitney test, was used. An analysis of the contingency tables was carried out using Fisher’s exact test. The two-tailed Student’s *t* test was used for comparisons between two groups. The statistical analyses were performed using Prism 5.0 for Windows software (GraphPad Software, San Diego, CA, USA).

## 3. Results

### 3.1. Sample Homogeneity Analyses

There are multiple risk factors that can influence the appearance and development of DR such as hypertension [26,27], dyslipidemia [11,28], HbA1c level [26], sex [29], inadequate glycemic control [30], higher body mass index [9], diabetes duration [26], higher intraocular pressure [10], homocysteine levels [31], and nephropathy [10]. So, we decided to evaluate the homogeneity in the population of diabetic patients with DR included in this study. Table 3 shows the results of the homogeneity study after the randomization of the sample. A statistical difference between the control group and the group treated with Pter is observed only in the DR stage category. The percentage of patients with moderate NPDR included in the group treated with Pter was higher than in the control group, indicating a worse initial ocular status or more advanced retinopathy (Table 3).

### 3.2. Pterostilbene Reduces Vascular Damage Progression in DR Patients

**Figure 1:**
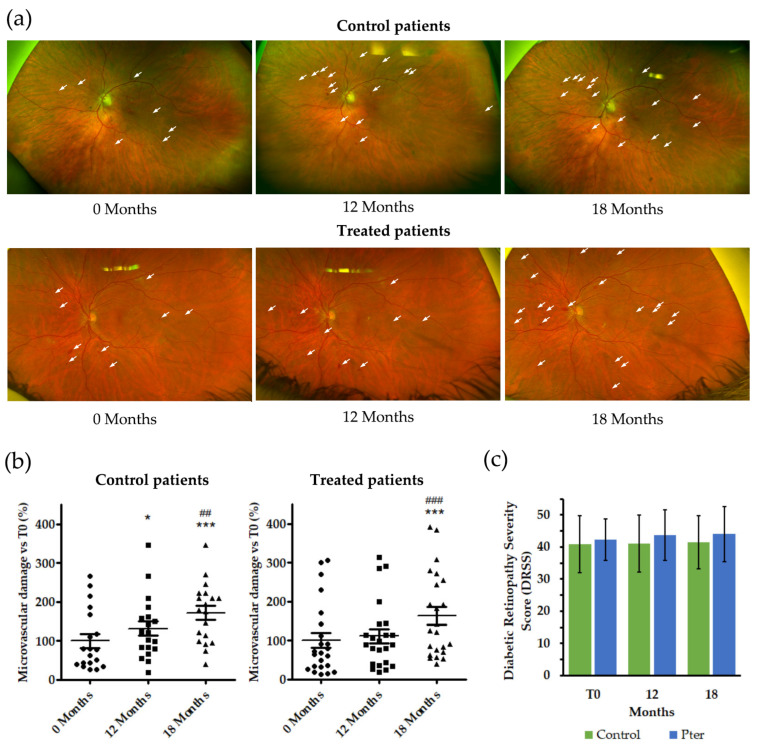
Pter reduces the development of microvascular lesions. The results were obtained at 0, 12, and 18 months. Control patients were treated with placebo. Pter was administered to the treated group only during the first 12 months. The last result was obtained 6 months later without treatment or placebo (18 months). (**a**) Eye fundus images of control and Pter-treated patients. The white arrows mark as an example some of the microvascular alterations evaluated. (**b**) Percentage of microaneurysms in non-treated (control) and treated patients vs. control average values at time 0 after 12 months of treatment and after 18 months (6 months later without treatment). For the evaluation, the fundus images of both eyes that were well focused were selected (n = 19 for control and n = 23 for treated). (**c**) Diabetic retinopathy severity score for both eyes of each patient (n = 52). Data are presented as mean ± S.D. Differences between groups were assessed using one-way ANOVA, followed by Tukey’s test. * *p* < 0.05; *** *p* < 0.001 versus 0 months. ^##^ *p* < 0.01, ^###^ *p* < 0.001 versus 12 months.

The appearance of microaneurysms, venous beading, and small intraretinal hemorrhages are one of the first observable symptoms in the development and progression of DR. In fact, the number of these microvascular lesions is one of the ETDRS staging criteria of the disease. As shown in Figure 1a,b, patients without specific therapy against DR (control patients) accumulate these lesions over time. This harmful effect of hyperglycemia on retinal tissue disappeared in patients treated with Pter for 12 months. The cessation of treatment with the polyphenol worsened the retinal status of the patients. In fact, an increase in the number of microvascular lesions was evident after suspending treatment for 6 months (18 months from the start of therapy) (Figure 1a,b). This precise analysis in DR progression contrasted with the clinical evaluation aimed in the pathology stage, since no differences were observed between the groups (Figure 1c). The ETDRS classification was created with the aim of having a simple and practical basis that would make it possible to predict the evolution of the disease and the treatment of DR before the appearance of severe vision loss. DR is a chronic disease with continuous progression; however, the clinical criteria used for staging the disease are based on discrete levels that group a set of damages. In our study, due to the slow progression of DR due to good glycemic control, an improvement or deterioration in the retina may be invisible because the alterations are insufficient to change the stage.

**Figure 2:**
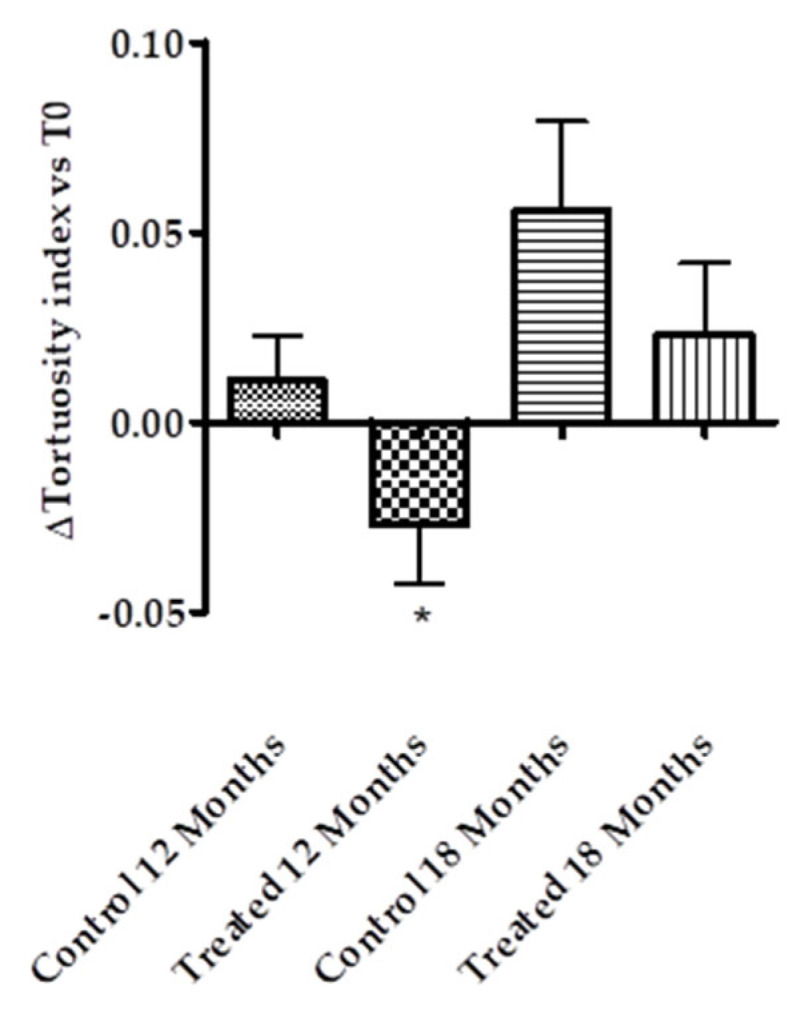
Pter reduces the vessel tortuosity index. Control and treated groups were treated daily with placebo or Pter for 12 months. Data were collected from the superior temporal retinal vein, superior nasal retinal vein, inferior temporal retinal vein, and inferior nasal retinal vein. The results represent the increment in the vessel tortuosity index after 12 months of treatment and 6 months later without Pter supplementation (18 months). The data are presented as the mean ± S.D. of 26 patients. Statistical analysis was performed using a Student’s *t* test. * *p* < 0.05 versus control vessel tortuosity index at the beginning of the study.

The effect as a vascular protector was also observed at the level of vascular tortuosity. Diabetes induced a continuous and progressive alteration in the retinal venous tortuosity in control patients; however, Pter was able to reduce the increment of tortuosity over time in the main retinal veins. This effect disappeared after 6 months without stilbene therapy (Figure 2).

### 3.3. Effects of Polyphenol Treatment on Diabetic Retinopathy Risk Factors

**Figure 3:**
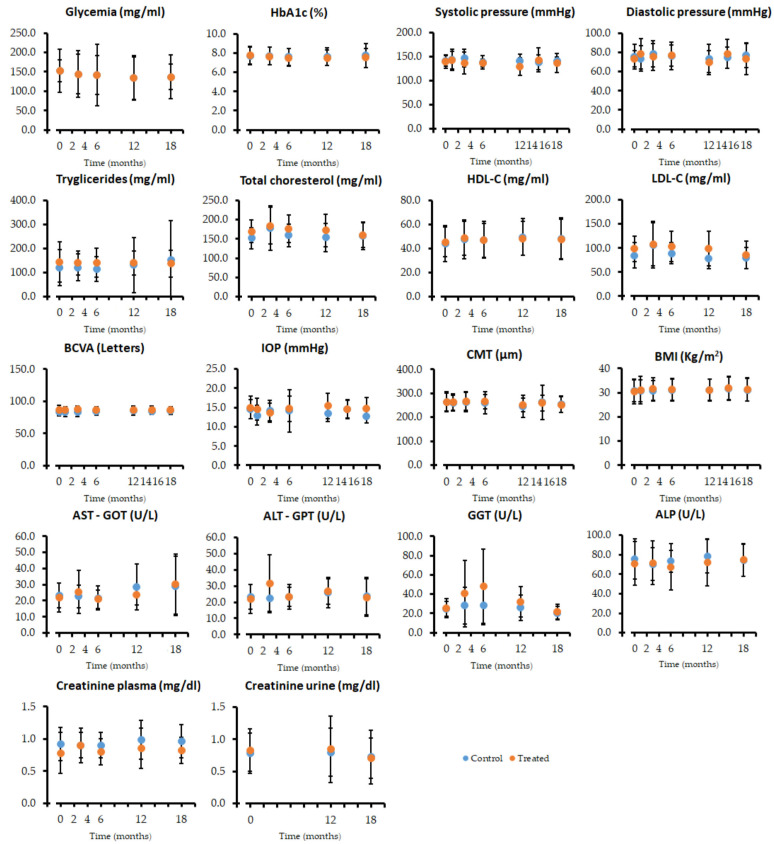
Analytical parameters to evaluate the effect of the treatment on the main risk factors and ocular, liver, and kidney function (n = 26).

In order to gain insights into the effects of polyphenolic treatment on the risk factors related to the development of DR, we analyzed glycemia, HbA1c, blood pressure, TGs, TC, HDL-C, LDL-C, and BMI, as well as ocular, liver, and kidney function. The levels of all these parameters remained unchanged over the time in which the study was developed (Figure 3).

### 3.4. Effects of Pterostilbene on Redox Status in Patients’ Blood

**Figure 4:**
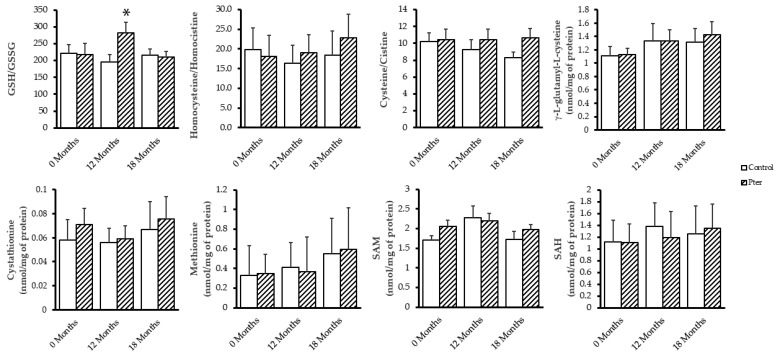
Blood redox status analyses. Molecules of transsulfuration pathway, GSH synthesis, and redox pairs were evaluated in patients’ blood. Data are presented as mean ± S.D. (n = 26). Statistical analysis was performed using Student’s *t* test. * *p* < 0.05 versus values obtained at the beginning of the study (0 months).

The patients’ blood tests showed an improvement in glutathione redox status, expressed as the GSH/GSSG ratio, after 12 months of treatment with the polyphenol (Figure 4). However, the evaluation of different components of the transsulfuration pathway, such as methionine, cystathionine, SAM, and SAH; the γ-glutamylcysteine levels, whose formation is the rate-limiting step in GSH biosynthesis; and the different redox pairs related with the oxidation state such as homocysteine/homocystine and cysteine/cystine did not suffer significant alterations over the time of the study with or without treatment (Figure 4).

### 3.5. Pterostilbene Protects Against Lipid Peroxidation Induced by Chronic Hyperglycemia

**Figure 5:**
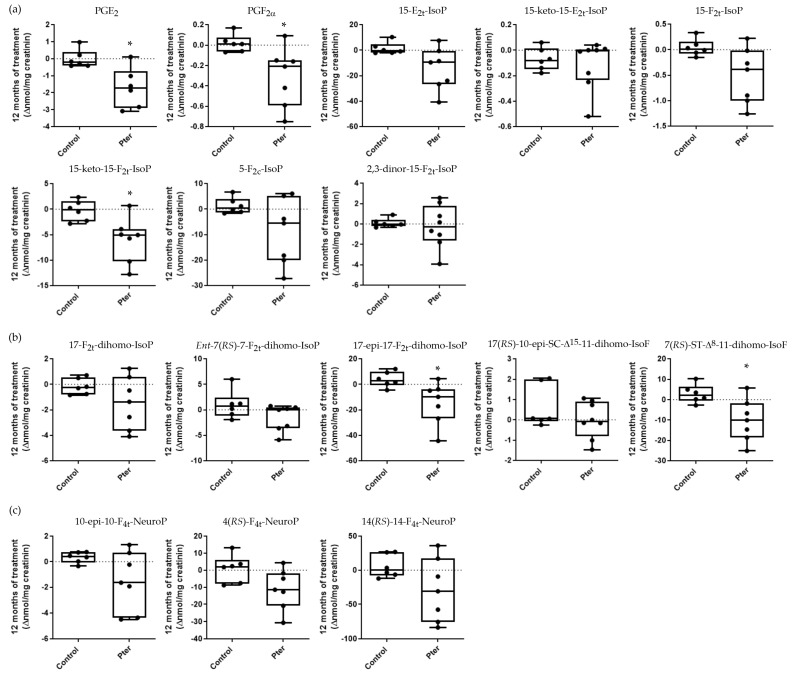
Box plots representing the variation in lipoperoxidation analytes in urine samples from control and treated patients after 12 months. Lipid peroxidation compounds were derived from (**a**) arachidonic acid, (**b**) adrenic acid, and (**c**) docosahexaenoic acid. The data represent the variation suffered by each patient in each parameter after 12 months of treatment. Boxes indicate the 1st and the 3rd quartiles, the average is shown as a black line, and whiskers mark the maximum and the minimum values. Statistical analysis was performed using the Mann–Whitney U test (n = 6 for control and n = 8 for treated; the determination was only carried out in the patients who delivered urine in the visits carried out in months 0, 12, and 18, without failing in any of them). The statistical difference is indicated as * < 0.05 vs. control.

**Figure 6:**
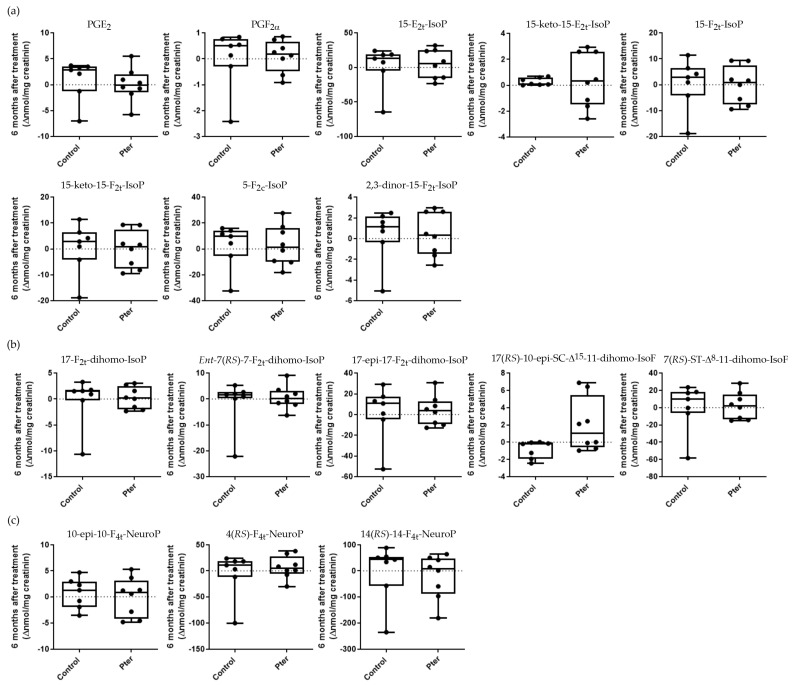
Box plots representing the variation in lipoperoxidation analytes in urine samples from control and treated patients after 18 months. Patients were treated with placebo or Pter (250 mg/day) for 12 months. Urine samples were collected 6 months later, after therapy was stopped (total of 18 months). Lipid peroxidation compounds were derived from (**a**) arachidonic acid, (**b**) adrenic acid, and (**c**) docosahexaenoic acid. The data represent the variation suffered by each patient in each parameter. Boxes indicate the 1st and the 3rd quartiles, the average is shown as a black line, and whiskers mark the maximum and the minimum values. Statistical analysis was performed using a Mann–Whitney U test (n = 6 for control and n = 8 for treated; the determination was only carried out in the patients who delivered urine in the visits carried out in months 0, 12, and 18, without failing in any of them).

Diabetes is a disease characterized by notable alterations in the homeostasis of the internal medium, beyond the development of hyperglycemia. Organ physiological changes induced by the disease are reflected in plasma and urine due to the continuous transport and mixing of the internal medium by the action of the circulatory system and the molecular exchange between interstitial fluid and plasma at tissue level. Recently, we showed that chronic hyperglycemia induces early lipid oxidation in the retina of experimental animals by 4-hydroxy-2-nonenal analyses [13]. The analysis of lipid peroxidation in depth allowed us to show how diabetes altered the oxidative state of derivatives of the arachidonic, adrenic, and docosahexaenoic acid in retinal, urine, and plasma samples of diabetic rabbits. Moreover, the daily subcutaneous administration of Pter was able to reverse these early oxidative alterations [12]. With the aim of delving into the beneficial effects of Pter in the treatment of DR, we analyzed the same lipid oxidation analytes in urine samples obtained from diabetic patients with DR. The analysis was performed in patients treated with polyphenol or placebo after 12 months of treatment (Figure 5) and 6 months later without administration of the stilbene (Figure 6). The antioxidant capability of Pter was shown in a reduced number of lipid oxidation analytes after 12 months of treatment. The protective effect of Pter was reflected in the decrease in the derivatives of arachidonic acid PGE_2_, PGF_2α_, and 15-keto-15-F_2t_-IsoP (Figure 5). In a similar way, the levels of the adrenic acid products 17-epi-17-F_2t_-dihomo-IsoP and 7(*RS*)-ST-Δ^8^-11-dihomo-IsoF were also reduced (Figure 5). The cessation of treatment for 6 months reversed the beneficial effects of the polyphenol. In fact, as shown in Figure 6, we found undetectable changes for the analytes derived from arachidonic, adrenic, and docosahexaenoic acids.

## 4. Discussion

Chronic hyperglycemia is the main cause of ocular neurovascular damage and preventable blindness in active people [32,33]. Progressive retinal damage appears slowly, and it is usually undetectable for patients in early stages of the pathology, which makes retinal neural lesions irreparable at the time of diagnosis and represents a point of no return. In this way, to break the advance of retinal damage is a scientific and medical priority. Oxidative stress is considered one of the main etiological factors in the development and progression of DR [5,7,12,13,14,15,16,17]. Exposure to light and the high metabolic activity of the retina produces continuous exposure to oxidative aggression. Under physiological conditions, the antioxidant machinery of this tissue is well adapted and maintains redox balance and cellular functionality. However, under the pathophysiological conditions induced by diabetes, the balance between the formation and elimination of oxidative species can be altered, generating oxidative stress. In this scenario, damage to proteins, DNA, and lipids is promoted, leading to cell death and potentially triggering retinal degeneration. Photoreceptors are cells especially sensitive to oxidative stress and lipid peroxidation due to the high polyunsaturated fatty acids (PUFA) content of their cell membranes [16]. Different nutraceuticals such as chrysin [34], resveratrol [35], epigallocatechin gallate [36] and curcumin [37] have demonstrated protector capabilities against free radicals in different retinopathies such as DR. Moreover, the potential benefits of oral antioxidant combinations have been demonstrated over longer periods of treatment and follow-up, with no significant changes generally observed in terms of BCVA or macular thickness [38]. The group of García-Medina et al. demonstrated a significant reduction in the percentage of patients progressing in the DR stage according to the International Clinical Diabetic Retinopathy Severity Scale after 60 months of supplementation with the formula Vitalux Forte^®^ (Vitamins C and E, lutein, zinc, copper, selenium, manganese, niacin, and carotene) compared to a placebo. They included type 2 diabetic patients with NPDR, but they did not specify the baseline DR stage of the patients included [39]. Sanz González et al. and Roig-Revert et al. demonstrated a delay in the progression of the ETDRS stage compared to placebo with the use of the commercial formula NutrofOmega^®^ (Vitamins C, D, B, and E, DHA, lutein, zeaxanthin, glutathione, hydroxytyrosol, zinc, copper, selenium, and manganese) after 18 and 38 months of treatment, respectively, in type 2 diabetics without DR or with mild NPDR [40,41]. Other trials in humans have demonstrated an early influence on functional retinal parameters after shorter follow-up periods, ranging from 6 to 14 months. The parameters analyzed included retinal sensitivity [42,43], contrast sensitivity [42,44], glare sensitivity [42], and macular pigment optical density [42]. The profile of these patients consisted of individuals with type 1 or type 2 diabetes and mild to moderate NPDR without DME, or with DME but without retinal thickening on OCT. These effects were observed with the use of lutein, the DiVFuSS^®^ formula (Vitamins D and E, lipoic acid, coenzyme Q10, omega-3 fatty acids, zeaxanthin, lutein, zinc, grape seed extract, resveratrol, pycnogenol, green tea leaf extract, and turmeric root extract), and a combination of desmin, troxerutin, *Centella asiatica*, and *C. melilotus* [42,43,44]. These findings suggest that antioxidants represent a valid prophylactic adjunct therapy in the early stages of DR, where anatomical damage is not excessive and there is no central macular thickening. However, the complex formulation of these nutraceuticals makes it difficult to determine the role of each compound by itself and its relative importance in preventing DR progression.

We have demonstrated that Pter has protective effects against DR development both in cellular and in vivo models [13]; however, its effects on DR patients are not clear. To study the potential benefits of Pter, patients suffering from DR were assigned to two different groups, placebo- (Control) or Pter-treated. Both groups showed similar demographic and homogeneous characteristics, as shown in Table 1. There was one statistical difference, since in the group of patients treated with Pter, the percentage of people with moderate NPDR was slightly higher than those with mild NPDR. This means that the initial state of the Pter-treated people was worse than that of the control population (Table 1). Pter was administered daily in two doses of 125 mg for 12 months. This dose of 250 mg/day has been demonstrated to be safe and effective both in animal models and clinical trials. For example, patients with hypercholesterolemia were treated with the polyphenol for an average of a 52-day duration without adverse effects [22]. Furthermore, healthy volunteers were treated with 450 mg of *Pterocarpus marsupium* extract for 14 days without showing toxicity symptoms [45]. In this regard, we did not observe any side reactions (Figure 3), nor did the patients report any gastrointestinal or other adverse outcomes throughout the study.

The Pter administration protocol reduced the formation of new retinal damage, as indicated by the number of microaneurysms, venous beading, intraretinal hemorrhages, and microvascular abnormalities. However, the slow but steady onset of retinal vessel damage reappeared after treatment removal and was detectable after 6 months without treatment (Figure 1a,b). The improvement observed in the treated patients was not reflected in a change in the staging performed by expert ophthalmologists (Figure 1c). The ETDRS scale used by clinicians to measure DR severity in patients is categorized into discrete steps. Specifically, the analysis of fundus stereographs establishes 13 levels of evolution of the disease that range from the absence of DR to the most advanced stages of PDR with vitreous hemorrhages and tractional retinal detachments [46,47]. This implies that minor improvements or deteriorations in the disease may go undetected, as they do not result in a change in stage on the ETDRS scale. For the present pilot clinical study, patients with mild to moderate NPDR were selected. As shown in Figure 1c, the stage of the patients treated with placebo or Pter did not change during the 18 months of the study. Given the slow progression of DR with good glycemic control, the study’s duration, and the cohort of patients with mild to moderate NPDR, these results are entirely expected. However, future research should focus on assessing diabetic patients without DR over a longer study period.

Obviously, good control over body homeostasis in all the risk factors that can contribute to the progression of the disease are beneficial and delay the evolution of the disease. Figure 3 represents both excellent control and the evolution of diabetes throughout the study of BMI, blood pressure, blood levels of glucose, HbA1c, and lipids (TGs, TC, HDL-C, LDL-C), as well as parameters related to ocular function (BCVA, IOP, CMT), liver function (AST-GOT, ALT-GPT, GGT, ALP), and kidney function (plasma and urine creatinine). Regardless of the existence or not of treatment, no significant changes were observed in any of the variables in the 18 months of the study. Previously, Hougee et al. showed that *Pterocarpus marsupium* extract administration (450 mg/day) for 14 days did not induce significant changes in hematology and blood chemistry values, including erythrocyte sedimentation rate, hemoglobin, hematocrit, white blood cell count, urea, creatinine, Na, K, Cl, GGT, ALP, bilirubin, AST-GOT, and ALT-GPT [45]. Our results show that a good control of diabetes together with Pter treatment delays the progression of DR, and also that polyphenol treatment is not capable of reversing the lesions developed in the retina and that are used in the ETDRS classification (Figure 1).

Data on the relationship between vascular tortuosity in the retina and DR progression has been controversial. In an Anglo-Scandinavian Cardiac Outcomes Trial (ASCOT) a sub-study found no significant differences in arterial tortuosity between diabetic and non-diabetic patients in a population of 711 individuals (159 with and 552 without diabetes) [48]. Analyses of 327 people (224 with diabetes and 103 non-diabetic controls) in another clinical study showed that diabetic patients had increased arteriolar and venular tortuosity compared to the healthy population. In addition, individuals with mild and moderate NPDR had greater arteriolar tortuosity than the diabetic population; however, they found no association between venular tortuosity and DR severity [49]. A population-based cross-sectional study of eye diseases in urban Malay adults found that diabetic individuals had less arteriolar tortuosity than healthy people [50]. In contrast, branch arterial tortuosity has been proposed as a specific indicator for the early detection of DR, as well as for the evaluation of the severity of DR [51]. These presented discrepancies are unknown but can be attributed to different factors such as baseline inclusion criteria or the performance of retrospective studies in different groups of patients without taking repeated measurements over time, and fundoscopy images resolution has been proposed as another discrepant reason [52]. However, although the method must be standardized so that it can have adequate diagnostic value, the future implementation of retinal blood vessel tortuosity has been suggested by different authors in the stratification of DR progression [52,53,54]. Quantitatively analyzed fundoscopy images allowed Lim et al. to report that the venular and arteriolar tortuosity of the great retinal vessels was associated with the progression of DR at one year of follow-up [53]. Cheung et al. showed that after 6 years of follow-up, retinal arteriolar tortuosity, venular branching angle, venular branching coefficient, arteriolar fractal dimension, and arteriolar caliber were associated with an increased risk of DR in type 2 diabetic patients [54]. Moreover, Klein et al. demonstrated that retinal venular tortuosity was associated with the incidence of PDR over a 5-year period [55]. In our study, the mean value of the retinal blood vessel tortuosity of the main retinal venules (superior temporal, inferior temporal, superior nasal, and inferior nasal) was reduced by polyphenol therapy (Figure 2). In concordance with the development of microvascular damage (Figure 1a,b), blood vessel tortuosity worsened significantly after 6 months without treatment (Figure 2).

Chronic hyperglycemia alters the redox status at a systemic level in diabetic patients. In an animal model, we were able to verify how both the oxidative stress induced in the retina and the early pathophysiological alterations developed in the organ by the effect of diabetes could be prevented by the action of Pter. So, the polyphenol decreased the neurotoxic effects on ganglion cells without improving the diabetic state and prevented both lipid and protein oxidation. In addition, the daily administration of Pter decreased the concentration of hydrogen peroxide in the retina and activated the enzymatic and non-enzymatic antioxidant machinery. Our results showed, as in other experimental models [56,57,58,59], the role played by Pter in the activation of NRF2 (nuclear factor erythroid 2-related factor 2) and its importance in the regulation of redox homeostasis [13]. In order to corroborate the capacity of Pter to regulate the systemic redox status in diabetic patients with DR, the levels of GSH and GSSG, as well as the main constituents of the transsulfuration pathway, were evaluated. GSH is a non-protein thiol essential for the regulation of body redox homeostasis. The transsulfuration pathway is a metabolic process that connects the methionine pathway with the production of cysteine, the limiting amino acid in the synthesis of GSH, thus contributing to the maintenance of the redox status. Furthermore, homocysteine accumulation has been associated with a decrease in insulin secretion, and the production of oxidative stress [60] has indeed been proposed as a biomarker in DR screening [31]. In our clinical study, we detected an improvement in the GSH/GSSG ratio in the blood of patients treated with Pter for 12 months compared to the patients with DR treated with placebo. In addition, after the administration of the polyphenol, a normalization of the ratio to the values of the untreated patients was observed (Figure 4). The rest of the parameters evaluated and compared to their oxidized counterparts did not suffer statistically significant variations detectable in blood between the control and treated patients (Figure 4). These results confirm Pter’s ability to modulate reactive oxygen species levels, for example, by enhancing antioxidant enzyme activity, as we previously demonstrated [13]. The observed change in the GSH/GSSG ratio is primarily attributable to a decrease in GSSG levels rather than an increase in GSH production.

In order to further study the antioxidant capacity of Pter, we studied the degree of quantifiable lipid peroxidation in urine samples. PUFAs are an essential component among the biomolecules of cell membranes for homeostatic maintenance and retinal function. In fact, the phospholipids of the cell membranes in the retina contain the highest tissue concentration of PUFAs in the body. Although the retina is an organ well adapted to physiological reactive oxygen species production, the oxidative stress produced by chronic hyperglycemia attacks PUFAs, which are susceptible to degradation and are a target for lipid peroxidation [17]. The lipid peroxidation of PUFAs in the retina is related to DR development [61] and the progression of the disease [62] and may be detected in the early stages of DR [12]. Lipid peroxidation worsens with the duration and severity of diabetes [63], induces retinal damage, such as the demise of pigment epithelial cells [64], alters the integrity of retinal neurotransmitter pools [65], and triggers inflammation and the development of retinal neovascularization [66,67]. Moreover, higher levels of lipid peroxidation have been demonstrated in patients suffering from DR than in those without retinal disease [68,69]. We demonstrated that a lipid peroxidation analysis of plasma and urine can reflect the oxidative status of the retina in an experimental animal model, and we postulated that this determination may also become a useful tool to determine the clinical interventions that patients should undergo [12]. In addition, the daily administration of Pter reduced the oxidation of docosahexaenoic acid (22:6n-3), arachidonic acid (20:4n-6), and adrenic acid (22:4n-6), PUFAs altered by oxidative stress in retinal pathologies [12]. Thus, although further research is needed to understand the correlation between lipid peroxidation and the reflection of retinal damage in plasma and urine, we set out to evaluate a set of derivatives of the oxidation of the three PUFAs found in the urine of the patients included in our study. The antioxidant capacity of Pter against lipid peroxidation was statistically significant for a small number of the oxidative derivatives of arachidonic acid and adrenic acid. Figure 5 shows how treatment with the polyphenol reduced the levels of the derivatives of arachidonic acid PGE_2_, PGF_2α_, and 15-keto-15-F_2t_-IsoP and the levels of the adrenic acid products 17-epi-17-F_2t_-dihomo-IsoP and 7(*RS*)-ST-Δ^8^-11-dihomo-IsoF in patients treated for one year compared to the control group. After 6 months without treatment, the protective effect disappeared, reaching similar values for all the derivatives studied (Figure 6). Applying strict patient selection criteria made it possible to have a more homogeneous sample, but it also made it difficult to include individuals in this study. Thus, due to the small number of patients recruited in the study, it was difficult to obtain significantly different values in the rest of the derivatives analyzed, and only a tendency for the values obtained in the treated patients to be reduced could be observed. Independently, the relationship between DR and the derivatives with evident differences has previously been established. Prostaglandin–endoperoxide synthase, known as cyclooxygenase (COX), catalyzes the biosynthesis of prostanoids from arachidonic acid derivatives such as PGE_2_ and PGF_2α_, important pro-inflammatory prostaglandins produced predominately by the isoform COX2 [70,71]. In addition, PGE_2_ and PGF_2α_ have been related to other pathophysiological effects of DR such as blood ocular barrier disruption, VEGF production, leukocyte migration, and vascular disfunction [71,72,73,74,75]. The quick metabolism from the circulation of the isoprostane 15-F_2t_-IsoP produces 15-keto-15-F_2t_-IsoP [76], a vasoconstrictor on the isolated thoracic aorta of rats [77]. Like PGE_2_ and PGF_2α_, 15-keto-15-F_2t_-IsoP is overexpressed both in the retina and blood of diabetic animals [12]. Moreover, the expression of COX2 isoforms and prostaglandin production are increased in the retinas of experimental diabetic animals and in high-glucose-treated retinal cells [13,78,79]; in fact, it has been suggested that the isoform COX2 has a dominant role in DR [80]. Our results show the capability of Pter to decrease the levels of these pro-inflammatory prostaglandins derived from the expression of COX2. Interestingly, this polyphenol has been shown to be a selective inhibitor of COX2 able to inhibit prostaglandin production in lipopolysaccharide-stimulated human peripheral blood mononuclear cells [45]. The oxidation of docosahexaenoic and adrenic acids has been found to occur in the central nervous system and retina [81,82,83,84,85], and indeed these have been considered biomarkers for neurological damage detection affecting the brain and retina such as for Alzheimer’s disease [81], age-related macular degeneration [86], or DR [12]. Here, we detected in urine lower lipid peroxidation products derived from the adrenic acids 17-epi-17-F_2t_-dihomo-IsoP and 7(*RS*)-ST-Δ^8^-11-dihomo-IsoF in treated patients (Figure 5), and together with arachidonic acid derivative analyses, these may be considered parameters to follow DR evolution under antioxidant therapies.

## 5. Conclusions

Despite the strict regulation of body homeostasis, DR progresses gradually in diabetic patients. Among the alterations that remain undetectable through traditional diagnostic methods are the slow and gradual development of microvascular lesions, changes in vascular tortuosity, and increased oxidative damage, parameters that can aid in monitoring disease progression in patients undergoing antioxidant treatment. Furthermore, the findings indicate the importance of initiating antioxidant therapies, such as Pter, at the time of diabetes diagnosis to help prevent retinal damage and DR development.

## Figures and Tables

**Table 1:** Baseline inclusion and exclusion criteria for patients to participate in the clinical study.

Inclusion Criteria	Exclusion Criteria
Patients aged between 50 and 76 years old	Any personal situation impeding following the study protocol
Type 2 diabetes *mellitus* presenting HbA1c ≤ 10.0% during the first visit	Active ocular inflammation or infection
Eyes with mild to moderate NPDR presenting non-clinically significant DME	A diagnosis of cataract with indication for surgery
Best corrected visual acuity (BCVA) ≥ 34 ETDRS letters	Macular structural damage impeding visual acuity improvement
No previous ocular surgeries, except for phacoemulsification at least 6 months prior to enrollment	Any kind of ocular surgery or intravitreal treatment during the 6 months prior to enrollment
No photocoagulation or anti-vascular endothelial growth factor (VEGF)/steroid intravitreal treatment for at least 6 months prior to enrollment	Presence of clinically significant DME
Signature of the inform consent document	Known hypersensitivity to fluorescein contrast
	Acute cardiovascular disease
	Significant hepatic, renal, or gastrointestinal disease
	Patients under treatment with thiazolidinediones or fibric acid

**Table 2:** Study protocol and variables measured at each visit.

Measurements and Actions	0 Month	1 Month	3 Months	6 Months	12 Months	15 Months	18 Months
	Visit 1 (T0)	Visit 2	Visit 3	Visit 4	Visit 5 (T1)	Visit 6	Visit 7 (T2)
Weight and height	x	x	x	x	x	x	x
Blood pressure	x	x	x	x	x	x	x
Best corrected visual acuity	x	x	x	x	x	x	x
Macular optical coherence tomography	x	x	x	x	x	x	x
Tonometry	x	x	x	x	x	x	x
Wide-field retinography	x	x	x	x	x	x	x
Intraocular pressure	x	x	x	x	x	x	x
Wide-field fluorescein angiography	x				x		x
Blood sample	x		x	x	x		x
Urine sample	x				x		x
Pter/placebo delivery	x	x	x	x			

**Table 3:** Demographic and initial characteristics of the study patients.

		DR	DR + Pter	*p* Value
Demographic parameters	Age (years)	65.00 ± 5.66	64.08 ± 5.04	0.857 (MW)
	Female (%)	20.00	21.43	0.593 (Fis)
	Male (%)	80.00	78.57	
Blood: Glucose	Glycemia (mg/dL)	123.20 ± 28.51	153.10 ± 55.46	0.171 (MW)
	HbA1c (%)	7.73 ± 0.99	7.74 ± 0.90	0.868 (MW)
Blood: Lipids	TG (mg/dL)	119.30 ± 73.95	142.60 ± 82.97	0.340 (MW)
	TC (mg/dL)	151.7 ± 26.98	169.50 ± 29.03	0.115 (MW)
	HDL-C (mg/dL)	43.87 ± 14.88	45.47 ± 12.13	0.520 (MW)
	LDL-C (mg/dL)	84.79 ± 26.33	98.46 ± 26.13	0.094 (MW)
Eye Function	BCVA (letters)	83.63 ± 6.73	86.43 ± 7.12	0.315 (MW)
	IOP (mm Hg)	14.54 ± 2.50	14.96 ± 2.94	0.646 (MW)
	CMT (µm)	264.30 ± 42.65	262.70 ± 36.53	0.809 (MW)
Liver Function	AST-GOT (U/L)	23.33 ± 7.76	21.87 ± 8.96	0.395 (MW)
	ALT-GPT (U/L)	23.33 ± 7.76	21.87 ± 8.97	0.933 (MW)
	γ-GT (U/L)	24.60 ± 7.53	25.42 ± 9.96	0.696 (MW)
	ALP (U/L)	75.53 ± 20.63	70.80 ± 22.40	0.604 (MW)
Kidney Function Creatinine	Plasma (mg/dL)	0.92 ± 0.26	0.81 ± 0.32	0.089 (MW)
	Urine (mg/dL)	0.78 ± 0.31	0.83 ± 0.33	0.581 (MW)
Obesity	BMI (kg/m^2^)	30.76 ± 4.48	30.49 ± 5.16	0.967 (MW)
DR Stage	Mild NPDR (%)	54	31	** 0.0016 (Fis)
	Moderate NPDR (%)	46	69	
Vascular alterations	VTI (U.A.)	0.24 ± 0.18	0.26 ± 0.21	0.593 (MW)
Arterial blood pressure	Systolic (mmHg)	141.80 ± 10.92	139.60 ± 14.45	0.6674 (MW)
	Diastolic (mmHg)	75.50 ± 13.12	73.10 ± 8.50	0.7664 (MW)
Oxidative parameters	GSH/GSSG (blood)	223.00 ± 115.9	232.10 ± 120.20	0.663 (MW)
	Methionine (nmol/mg of protein)	0.87 ± 1.69	0.81 ± 1.93	0.616 (MW)
	SAM (nmol/mg of protein)	1.72 ± 0.40	2.05 ± 0.56	0.086 (MW)
	SAH (nmol/mg of protein)	1.11 ± 1.41	1.10 ± 1.12	0.6620 (MW)
	Homocysteine (nmol/mg of protein)	0.64 ± 0.30	0.65 ± 0.31	0.678 (MW)
	Homocystine (nmol/mg of protein)	0.05 ± 0.03	0.05 ± 0.04	0.678 (MW)
	Cystathionine (nmol/mg of protein)	0.06 ± 0.06	0.09 ± 0.05	0.082 (MW)
	Cysteine (nmol/mg of protein)	27.56 ± 11.17	30.29 ± 13.43	0.646 (MW)
	Cystine (nmol/mg of protein)	2.71 ± 0.61	2.58 ± 0.63	0.419 (MW)
	γ-glutamylcysteine (nmol/mg of protein)	1.11 ± 0.52	1.12 ± 0.37	0.945 (MW)
Lipid peroxidation (ng/mg of creatinine)	PGE_2_	1.92 ± 1.03	3.31 ± 2.66	0.1260 (MW)
	PGF_2α_	0.25 ± 0.11	0.41 ± 0.33	0.2366 (MW)
	15-E_2t_-IsoP	14.21 ± 10.04	20.09 ± 13.65	0.4025 (MW)
	15-keto-15-E_2t_-IsoP	0.06 ± 0.06	0.19 ± 0.31	0.6650 (MW)
	15-F_2t_-IsoP	0.46 ± 0.22	0.74 ± 0.52	0.2855 (MW)
	15-keto-15-F_2t_-IsoP	4.43 ± 2.68	7.75 ± 5.90	0.1260 (MW)
	2,3-dinor-15-epi-15-F_2t_-isoP	1.27 ± 0.76	1.71 ± 1.22	0.3408 (MW)
	5-F_2t_-IsoP	8.73 ± 3.74	13.63 ± 10.28	0.4357 (MW)
	7(*RS*)-ST-Δ^8^-11-dihomo-IsoF	10.22 ± 5.28	15.55 ± 9.58	0.1410 (MW)
	17(*RS*)-10-epi-*SC*-Δ^15^-11-dihomo-IsoF	0.47 ± 0.81	0.46 ± 0.73	0.6236 (MW)
	17-epi-17-F_2t_-dihomo IsoP	10.74 ± 6.44	18.35 ± 14.98	0.2366 (MW)
	17-F_2t_-dihomo IsoP	1.77 ± 0.77	2.52 ± 1.56	0.3708 (MW)
	Ent-7(*RS*)-7-F_2t_-dihomo IsoP	1.62 ± 1.13	2.81 ± 2.37	0.2602 (MW)
	14(*RS*)-14-F_4t_-NeuroP	33.17 ± 15.15	54.57 ± 40.56	0.3123 (MW)
	4(*RS*)-F_4t_-NeuroP	13.46 ± 9.12	18.52 ± 14.41	0.5444 (MW)
	10-epi-10-F_4t_-NeuroP	1.81 ± 1.08	2.98 ± 2.55	0.3123 (MW)
	Neuroprostanes	0.12 ± 0.14	0.08 ± 0.009	0.5444 (MW)
	Isoprostanes	0.11 ± 0.13	0.07 ± 0.02	0.9770 (MW)
	Neurofurans	0.10 ± 0.12	0.07 ± 0.01	0.8399 (MW)
	Isofurans	0.04 ± 0.05	0.03 ± 0.005	0.2855 (MW)

ALP: Alkaline phosphatase; ALT-GPT: alanine amino transferase/glutamate pyruvate transaminase; AST-GOT: aspartate aminotransferase/glutamic oxaloacetic transaminase; BCVA: best corrected visual acuity; BMI: body mass index; CMT: central macular thickness; γ-GT = gamma-glutamyl transferase; GSH/GSSG: glutathione/glutathione disulfide; HbA1c: hemoglobin A1c (glycated hemoglobin, glycosylated hemoglobin); HDL-C: high-density lipoprotein cholesterol; IsoF: isofuran; IsoP: isoprostane; IOP: intraocular pressure; LDL-C: low-density lipoprotein cholesterol; NeuroP: neuroprostane; NPDR: non-proliferative diabetic retinopathy; PG: prostaglandin; SAH: S-adenosyl-L-homocysteine; SAM: S-adenosyl-L-methionine; TC: total cholesterol; TG: triglyceride; VTI: venous tortuosity index. Data are presented as mean ± S.D. Statistical differences between control (DR) and treated patients (DR + Pter) diagnosed with NPDR were analyzed by a non-parametric test, the Mann–Whitney test (MW). Fisher’s exact test (Fis) was conducted for comparison of sex between DR and DR + Pter. This test was also performed to compare the grade of NPDR between DR and DR + Pter (** *p* < 0.01).

## Data Availability

The data presented in this study are available within the article.
